# A Multilocus Nanopore eDNA Workflow for Red Algal Diversity Assessment in an Algal Reef System

**DOI:** 10.1002/ece3.74209

**Published:** 2026-08-23

**Authors:** Silvia Fontana, Wun‐Ruei Chang, Shao‐Lun Liu

**Affiliations:** ^1^ Department of Life Science & Center for Ecology and Environment Tunghai University Taichung Taiwan

**Keywords:** algal reef, crustose coralline algae, environmental DNA, multilocus metabarcoding, Oxford Nanopore sequencing, Rhodophyta

## Abstract

Environmental DNA (eDNA) metabarcoding is a powerful tool for rapid biodiversity assessment through high‐throughput sequencing of taxonomically informative genetic markers. However, the use of multiple high‐resolution genetic markers in red algae (Rhodophyta) is often constrained by the short‐read capacity of widely used platforms. To bridge this gap, we developed and evaluated a multilocus eDNA workflow optimized for red algal diversity using Oxford Nanopore Technologies (ONT) long‐read sequencing. We targeted four genetic regions (*rbc*L, *rpoC1*, *psb*A, and COI‐5P) and applied the workflow to one composite water sample and one composite sediment sample per site from two contrasting coastal habitats in northwestern Taiwan: a high‐reef algal site (Yongan) primarily concreted by crustose coralline algae (CCA) and a low‐reef sand‐based site (Potou). The ONT workflow enabled commonly used red algal markers with different amplicon lengths, ranging from approximately 350 to 850 bp, to be sequenced within a single pooled run. This approach simultaneously recovered informative red algal assemblages. Marker comparison showed that although *rpoC1* yielded the highest OTU (Operational Taxonomic Unit) richness, broad taxonomic and phylogenetic coverage required a multilocus strategy using *rbc*L, *rpoC1*, and *psb*A, whereas COI‐5P was not informative for red algal recovery in our pilot workflow. Rarefaction analysis indicated that sequencing depth was sufficient to recover most of the detectable red algal ASVs within most libraries, although low red algal read numbers limited *psb*A recovery in both Potou libraries, especially in sediment. Our results also highlight a methodological caveat where turf and frondose algae, rather than CCA, dominated the recovered red algal reads. This result may reflect differences in eDNA shedding among different algal growth forms. Overall, our study supports the use of long‐read multilocus ONT strategies for future red algal biodiversity surveys, while highlighting the need for marker‐specific interpretation, improved reference databases, and calibration against traditional surveys.

## Graphical abstract

This study evaluates an Oxford Nanopore Technologies long‐read sequencing workflow using a multilocus eDNA approach to assess red algal (Rhodophyta) biodiversity in coastal waters. Results demonstrate that while a combination of *rbc*L, *rpoC1*, and *psb*A markers (but not COI‐5P) provides the most comprehensive taxonomic coverage, eDNA signals are heavily influenced by algal turnover rates and shedding dynamics, often underrepresenting slow‐growing crustose coralline algae compared to conventional visual surveys.

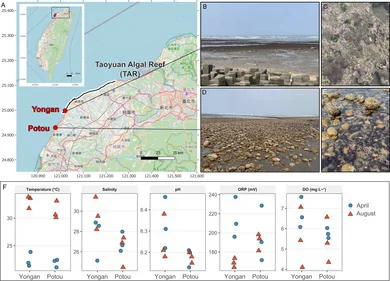

## 1 Introduction

Environmental DNA (eDNA) broadly refers to DNA that can be extracted directly from environmental samples (e.g., water, sediment, soil, or air) without first isolating target organisms. It includes both cellular material (e.g., shed cells or tissues) and extracellular DNA released after cell death and breakdown (Taberlet et al. 2012). eDNA metabarcoding is a technique in which selected genetic markers are amplified from an eDNA sample and sequenced in parallel using high‐throughput sequencing. The resulting sequences are compared with reference databases to detect multiple taxa simultaneously. This approach enables the detection of inconspicuous, rare, and morphologically cryptic taxa and can recover a broader set of taxa than visual surveys or single‐specimen barcoding, facilitating rapid, large‐scale community‐level biodiversity assessments (Ficetola et al. 2008; Thomsen and Willerslev 2015; Liu et al. 2020).

Interpreting the outcome of eDNA metabarcoding, however, requires accounting for two main hurdles: the physical eDNA dynamics in the environment and the genetic limitations of taxonomic resolution. In the former case, it has been widely known that environmental conditions influence DNA persistence. For instance, eDNA generally degrades faster at higher temperatures and under high‐UV conditions, whereas colder, low‐UV environments prolong its persistence (Strickler et al. 2015). Detection also depends on fragment length, as shorter eDNA fragments may persist longer and be recovered more frequently (Suter et al. 2023). In addition, transient biological material and eDNA from nonresident organisms, such as drifting propagules, cells, or tissue fragments, can produce false positives, whereas degradation or dilution can yield false negatives (Darling and Mahon 2011; Yates et al. 2023). To reduce such detection errors, eDNA studies commonly combine conventional survey calibration with standardized analytical pipelines and best‐practice guidelines to minimize artifacts and misassignments (Veron et al. 2023; Hakimzadeh et al. 2024). Beyond these detection‐related challenges, eDNA metabarcoding is also limited by taxonomic resolution. Reliance on a single short genetic marker can restrict taxonomic coverage and reduce species‐level discrimination, especially in diverse or closely related groups. Multilocus approaches can help address this limitation by broadening taxonomic recovery and reducing marker‐specific false absences across different communities, as shown for soil, marine, and algal assemblages (Drummond et al. 2015; Zhang et al. 2023; Borer et al. 2025). While multilocus strategies effectively expand taxonomic breadth, long‐amplicon sequencing provides a complementary advantage by increasing the sequence information (or capturing more genetic variation) per locus.

Rhodophyta represents the most species‐rich lineage of macroalgae in coastal ecosystems worldwide, accounting for more than half of recognized seaweed species (Guiry and Guiry 2021). This diversity makes high taxonomic resolution essential for meaningful biodiversity assessment. From a practical point of view, algal eDNA metabarcoding studies, including those reporting Rhodophyta, often rely on short regions of universal eukaryotic markers. For example, studies often target a single short fragment of the 18S rRNA gene, such as the V4 or V9 regions, to characterize broad eukaryotic assemblages and infer macroalgal composition. However, these regions often provide only higher‐level taxonomic resolution and may fail to resolve closely related species (Ørberg et al. 2022; Kezlya et al. 2023; Buhari et al. 2025; Canvin et al. 2026; Pearman et al. 2025). By contrast, the most extensive and taxonomically resolved reference databases for red algae are based mainly on COI‐5P and *rbc*L, two markers widely used in red algal DNA barcoding (Saunders 2005; Saunders and Moore 2013). Similarly, the plastid *psb*A marker is also widely used for species delineation in specific lineages, such as coralline algae (Broom et al. 2008; Anglès d'Auriac et al. 2019). More recently, *rpoC1* has emerged as a promising candidate marker for the phylogenetic community ecology of red algae because, compared with other commonly used markers, its gene tree more closely matched the phylogeny inferred from complete plastid genomes, and it was successfully amplified across a broad taxonomic range (Zhan et al. 2020). Thus, the markers best suited for red algal identification and biodiversity surveys are often longer than the short amplicons commonly used in eDNA metabarcoding, resulting in a mismatch between red algal taxonomic needs and standard sequencing approaches. Oxford Nanopore Technologies (ONT) provides a cost‐effective way to potentially address this gap by enabling long‐amplicon sequencing, including full or near‐full barcode regions. Recent applications of ONT to eDNA metabarcoding of marine vertebrates have shown improved species‐level resolution with longer amplicons (Doorenspleet et al. 2025). Although ONT historically had higher raw error rates than short‐read platforms, recent advances in chemistry, basecalling, and consensus‐based bioinformatics now enable accurate community profiling for markers such as 16S and COI‐5P (Zhang et al. 2023; Hebert et al. 2025). Therefore, ONT provides a practical and promising platform for multilocus red algal eDNA metabarcoding using longer, taxonomically informative markers.

Among red algal groups where cryptic diversity and encrusting growth forms hinder visual surveys, crustose coralline algae (CCA; Corallinophycidae, Rhodophyta) are especially challenging. CCA are nonarticulated, calcified red algae that form encrusting thalli on hard substrates from the intertidal to deeper waters and occur globally from tropical to polar regions (Woelkerling 1988). They create complex coastal habitats (e.g., Jardim et al. 2025), and are one of major producers of coastal carbonate in tropical and temperate systems (e.g., McCoy and Kamenos 2015; Cornwall et al. 2023). In several temperate and subtropical systems, including Mediterranean coralligenous formations and the Taoyuan Algal Reef (TAR) of northwestern Taiwan, CCA act as primary bioconstructors where scleractinian corals are sparse or secondary (e.g., Bosence 1983; Ballesteros 2006; Liou et al. 2017; Zhan et al. 2026). Their ecological importance, cryptic diversity, and difficulty of visual identification make CCA an informative focal group for evaluating ONT‐based eDNA workflows.

The TAR, on Taiwan's northwestern coast, is a subtropical CCA‐built algal‐reef complex and one of the world's largest subtropical intertidal algal‐reef systems (~27 km long and up to 450 m wide) (Liou et al. 2017). It is bordered by nonreefal sandy and cobble shores that provide nearby contrasting coastal habitats. Our prior work established a baseline inventory of CCA at TAR through the large‐scale DNA barcoding of nearly 800 specimens (Zhan et al. 2022). This labor‐intensive and costly approach revealed underappreciated cryptic diversity within the Sporolithales, Hapalidiales, and Corallinales that is often overlooked by morphology‐based surveys. This extensive specimen‐based dataset positions the TAR as an ideal testing ground for evaluating more efficient, high‐throughput alternatives. Multilocus long‐read eDNA metabarcoding, therefore, provides a potential tool for rapid red algal biodiversity assessment, but its effectiveness for complex groups such as coralline algae is best evaluated in systems with existing specimen‐based reference data.

To date, the use of multilocus long‐read sequencing for red algal eDNA remains largely unexplored. Here, we developed and evaluated a multilocus ONT eDNA workflow for assessing red algal diversity, with particular attention to CCA in the TAR. We targeted four genetic regions widely used for this group: three plastid loci, *rbc*L, *rpoC1*, and *psb*A, and one mitochondrial locus, COI‐5P. By integrating sample processing, amplification, long‐read sequencing, and marker‐specific bioinformatic filtering, we aimed to improve the recovery of informative red algal reads and to identify marker‐specific biases and nontarget amplification. We applied the workflow to water and sediment composite samples from two nearby contrasting habitats: Yongan, a high‐reef site within the TAR with a well‐developed CCA‐dominated algal reef, and Potou, a low‐reef site a few kilometers to the south, dominated by sandy and cobble substrates. This application also provided a preliminary comparison with the existing specimen‐based CCA baseline from the TAR. Overall, this pilot study compares marker performance, describes red algal recovery from the sampled water and sediment composites, and identifies methodological caveats for future multilocus ONT eDNA surveys of Rhodophyta.

## 2 Materials and Methods

### 2.1 Sampling Sites and Environmental Characterization

**FIGURE 1:**
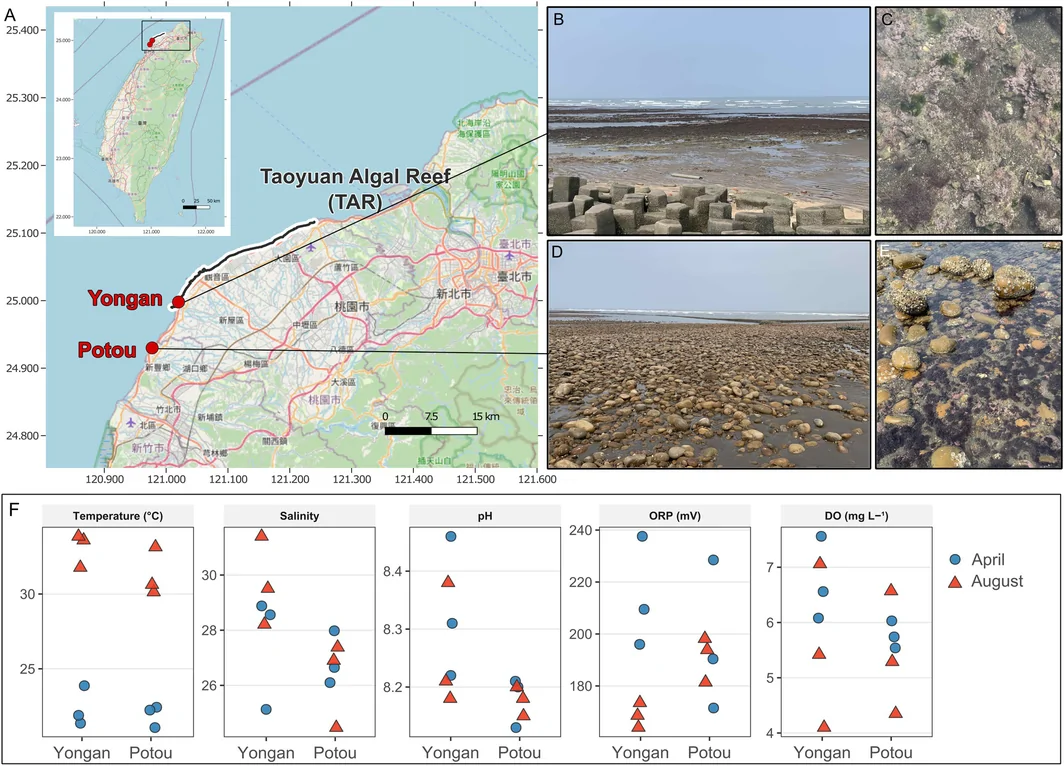
Environmental background of the two sampling sites. (A) Map of northern Taiwan showing the Yongan and Potou sampling sites and the extent of the Taoyuan Algal Reef (TAR). (B–E) Habitat contrasts between the two sites. (B) Yongan, showing high reef presence. (C) Yongan substrate, showing crustose coralline algae (CCA) and turf algae. (D) Potou, characterized by sand and scattered rocks. (E) Rock substrate at Potou, with no visible CCA. (F) In situ seawater parameters. Measurements were taken at Yongan and Potou on three dates in April 2023 (during biological sampling) and three dates in August 2023. Points show individual daily measurements. Panels show water temperature, salinity (PSU), pH, oxidation–reduction potential (ORP, mV), and dissolved oxygen (DO, mg L^−1^). Group means ± SD are reported in Table S3.

Environmental DNA (eDNA) samples were collected from seawater and sediment at two locations: Yongan (24.997868, 121.019980) and Potou (24.929904, 120.977300), approximately 8 km apart along the northwestern coast of Taiwan (Figure 1A). Yongan lies within the Taoyuan Algal Reef (TAR) and is dominated by reef habitat (Figure 1B,C), whereas Potou, immediately south of the TAR, is characterized primarily by sandy substrate with scattered cobbles (Figure 1D,E). All samples were collected on 25 April 2023, near the peak of the spring algal reproductive season.

At the time of sampling, water temperature, salinity, pH, oxidation–reduction potential (ORP), and dissolved oxygen (DO) were measured in situ with a HANNA multiparameter handheld water quality meter (model HI98194; Hanna Co., USA). Environmental parameters were also recorded at both sites on April 13, 22, and 25, and on August 9, 23, and 28, to provide descriptive environmental context for the two sites. For each month, standardized contrasts between Yongan and Potou were summarized using Hedges' *g*, calculated with the *effsize* package in R (R Core Team 2014). Given the limited number of observations, these effect sizes were used only as descriptive background and not as inferential evidence of ecological differences between the sites.

### 2.2 Sample Collection and DNA Extraction

At each site, water and sediment were collected from three positions 100 m apart along the shore and pooled to form one composite water sample and one composite sediment sample. No specific collection permit was required for the collection of seawater and surface sediment at these two sites. The three positions therefore represented subsamples of a single composite and did not constitute independent biological replicates. Accordingly, comparisons between sites and between water and sediment were evaluated descriptively. For water, three 1 L surface‐water samples were combined, mixed thoroughly, divided into two 500 mL aliquots, and supplemented with 500 μL proteinase K (1000 mg/L). For sediment, 50 g of surface sediment from the upper 1 cm was collected from the same three positions, pooled, homogenized, and partitioned into two 10 g aliquots. Each aliquot was transferred to a 50 mL centrifuge tube and immediately treated with 20 mL of RNAlater. Both water and sediment aliquots were transported to the laboratory in a chilled icebox within 2 h. Water samples were immediately filtered through 0.22 μm membrane filters. Sediment samples were centrifuged to remove the RNAlater supernatant. The remaining 10 g of sediment was then homogenized in a 1:1 (v/v) ratio with a preheated phosphate‐buffered solution (80°C, 1.97 g of NaH_2_PO₄ and 14.7 g of Na_2_HPO₄ per L of sterile water; Geraldi et al. 2020), supplemented with 20 μL proteinase K. Homogenization was performed for 30 min using a rotary suspension mixer (SM‐3000‐500, ZGene Biotech Inc., Taiwan). Following homogenization, the mixture was centrifuged, and the resulting supernatant was filtered through 0.22 μm membranes. All membrane filters were subjected to DNA extraction on the same day as sampling. Total DNA from both water and sediment filters was extracted with the DNeasy PowerSoil Pro Kit (Qiagen, Hilden, Germany).

### 2.3 Optimization of Sediment eDNA Extraction

Because initial direct DNA extractions from sediment did not yield amplifiable PCR products, we refined the sediment processing protocol through preliminary trials (see below for details). This troubleshooting is consistent with recent work showing that DNA recovery from marine sediments can be affected by mineral adsorption, lysis/desorption conditions, and co‐extracted PCR inhibitors (Gande et al. 2024). In our initial experiments, sediment treated with RNAlater and stored at −80°C prior to extraction did not yield visible PCR products when DNA was extracted directly from the sediment using the PowerSoil kit. Fresh direct extraction from RNAlater‐preserved sediment produced amplifiable DNA, but amplification from the same extracted DNA became unsuccessful after short‐term one‐ or two‐week storage at −20°C. We therefore tested a modified workflow in which sediment was homogenized in phosphate‐buffered solution, supplemented with proteinase K, centrifuged, and the supernatant was filtered onto membranes before DNA extraction. This membrane‐based workflow produced amplifiable DNA after storage of the extracted DNA at −20°C for more than one month. As an additional storage check, membranes prepared with the final protocol and stored at −80°C also yielded amplifiable DNA after one month, although freshly prepared membranes were used for the Nanopore sequencing libraries reported here.

### 2.4 Library Preparation and Sequencing

Four primer sets were used to amplify the target regions: three plastid genes (*rbc*L, *psb*A, and *rpoC1*) and one mitochondrial gene (COI‐5P). All primer sets were designed to preferentially amplify red algae: the *psb*A primer set was specifically developed for crustose coralline algae (CCA), whereas the *rbc*L, *rpoC1*, and COI‐5P primer sets target red algae more broadly (Saunders 2005; Saunders and Moore 2013; Anglès d'Auriac et al. 2019; Zhan et al. 2020). The *rpoC1* primer set was developed in our laboratory (Zhan et al. 2020). PCR was performed following the recommended protocols for each primer set (Table S1). Amplicon lengths were approximately 850 bp (*rbc*L), 350 bp (*psb*A), 600 bp (*rpoC1*), and 700 bp (COI‐5P).

PCR amplicons were prepared for high‐throughput sequencing as follows. Each PCR product was end‐repaired and A‐tailed using the KAPA HyperPrep Kit (Roche), followed by ligation with DNA ligase from the same kit. Each sample was quantified with a Qubit fluorometer, and barcodes were added with the Native Barcode Kit 24 V14 (Oxford Nanopore Technologies, ONT), following the manufacturer's instructions. Barcoded amplicons were purified using KAPA HyperPure magnetic beads (Roche), requantified, and pooled into a library. As shorter DNA fragments are typically sequenced more efficiently on Nanopore platforms, the four amplicons (850, 700, 600, and 350 bp) were pooled in molar ratios that were inversely proportional to their fragment lengths to reduce sequencing depth bias. Final molar amounts were calculated using Promega's Biomath Calculator (https://worldwide.promega.com/resources/tools/biomath/), resulting in a pool of 200 fmol in 65 μL. Sequencing adapters from the ONT kit were then ligated to the pooled library, which was loaded onto a MinION R10.4 flow cell (FLO‐MIN14, ONT).

A total of 16 environmental libraries were prepared. Eight libraries were generated for each of the two locations as follows: *rbc*L—water sample, *rbc*L—sediment sample, *rpoC1*—water sample, *rpoC1*—sediment sample, *psb*A—water sample, *psb*A—sediment sample, COI‐5P—water sample, and COI‐5P—sediment sample. In addition, one negative control library was prepared and sequenced as described below. Basecalling was performed with MinKNOW version 24.06.05.

### 2.5 Contamination Control

Contamination was identified as a major concern during initial testing. To monitor potential contamination, four no‐template PCR controls were prepared, one for each marker, using the corresponding primer pair and the same PCR reagents and conditions as the environmental samples, but without template DNA. No visible bands were observed for any of the controls following gel electrophoresis. The four control reactions were subsequently pooled and underwent the same product purification, barcoding, and library preparation procedures as the environmental libraries. The resulting negative control library was sequenced as a separate barcoded library. To minimize potential cross‐contamination, electrophoresis gels of the environmental samples were also visually inspected, and only samples without visible background noise or nonspecific fragments were advanced to the next step. Furthermore, all eDNA procedures were performed in a laminar flow chamber in a dedicated clean area using filtered tips, and different samples were processed on separate days.

### 2.6 Bioinformatic Analysis

Bioinformatic analyses followed a three‐step workflow. First, raw Nanopore reads from each library were demultiplexed, quality‐ and length‐filtered, and trimmed to remove barcodes, primer regions, and low‐quality bases. Second, denoised Amplicon Sequence Variants (ASVs) were inferred separately for each marker, filtered by length and orientation, and screened to remove nontarget sequences and contaminants. Third, processed reads from each library were mapped back to the curated ASV sets to generate library‐by‐ASV count tables and relative‐abundance profiles for downstream community analyses. Each analytical step is detailed below.

For the first step, data were demultiplexed with MinKNOW software to separate reads by barcode (one library per barcode). For each library, all demultiplexed FASTQ files were concatenated into a single FASTQ file prior to downstream processing. Read quality and length were assessed visually with NanoPlot (De Coster et al. 2018). Correct sample assignment was confirmed by screening reads for the presence of forward or reverse barcodes, allowing up to two mismatches (Hebert et al. 2025). Residual barcodes were removed by trimming sequences before the forward barcode and after the reverse barcode, allowing up to two mismatches. Because PCR primer regions often accumulate substitution errors during amplification (Schirmer et al. 2015), primers could not be reliably detected in our data even with a two‐mismatch allowance. To ensure their complete removal, 25 bp were trimmed from both the 5′ and 3′ ends of every read. After trimming, a first length filter was applied: reads were trimmed to a maximum of 850 bp, and those shorter than 200 bp were discarded. Previous studies (Hsieh et al. 2018; Hebert et al. 2025) and quality profiles generated with NanoPlot for our dataset (De Coster et al. 2018) show that read quality declines sharply in very long amplicons; therefore, the 850 bp ceiling eliminates most low‐quality over‐length reads while preserving the full‐length *rbc*L amplicon, the longest target. Reads shorter than 200 bp were excluded because they provide insufficient sequence even for the shortest amplicon, *psb*A. Reads with an average quality score below 20 (i.e., 1% error rate) were discarded. To calculate the average quality of a read, each base's Phred quality score (Qi) was converted to an error probability, the probabilities were averaged, and the result was converted back into a Phred score (Ewing and Green 1998), using the formula:

$$Q_{\mathrm{avg}}=-10\log_{10}\left(\frac{1}{n}\sum_{i=1}^{n}10^{-\frac{Q_{i}}{10}}\right)$$

where *Q*_avg_ is the average quality score of the read, *Q*_*i*_ is the Phred quality score at base *i*, and *n* is the number of bases in the read. This approach ensures that the average quality score accurately reflects the read's expected error rate. All read processing was performed using a custom Python script.

For the second step, to generate the set of unique, denoised sequences (Amplicon Sequence Variants, ASVs), we first combined all libraries corresponding to the same amplicon, resulting in four datasets (*rbc*L, *rpoC1*, *psb*A, and COI‐5P), each containing data from four libraries (Yongan and Potou, water and sediment samples). Unique sequence calling and denoising were performed with USEARCH (“fastx_uniques” followed by “unoise3” functions) (Edgar 2010), which clusters identical reads, removes sequencing noise, and eliminates potential chimeras. Only unique sequences supported by at least 8 reads were retained for denoising, following the software's recommendations. A marker‐specific length filter was applied: ASVs shorter than 75% of the median sequence length were discarded, as these were considered too short to provide reliable information for each marker. Because denoised ASVs may occur in either orientation, reverse‐complemented sequences were re‐oriented using local BLAST against a representative algal sequence of the intended marker. This local BLAST step also addressed the problem of spurious ASVs resulting from nontarget amplifications: unlike true target sequences, nontargets either fail to align or produce poor alignments to the representative sequence. ASVs yielding BLAST bitscores ≥ 100 against the representative sequence were retained, while those below this threshold were considered nontarget. In addition, ASVs with BLAST bitscores ≥ 200 against an *rpoC1* reference from *Caulacanthus okamurae*, identified during preliminary analyses as a recurring contaminant, were classified as *rpoC1* contaminants. Thus, local BLAST served both to standardize sequence orientation and to identify and remove nontarget ASVs. To further confirm nontarget amplifications, ASVs were also queried with BLASTn against the NCBI GenBank database, and suspect hits were manually examined. ASVs were aligned and trimmed to a uniform length. Because some SNPs fell within the trimmed regions, redundant ASVs that became identical after trimming were discarded. Finally, ASVs containing premature stop codons or frameshift mutations were removed. Except for the initial denoising performed with USEARCH (see above), all subsequent data processing was performed using custom Python scripts developed for this study.

For the final step, processed reads from each library (Yongan water, Yongan sediment, Potou water, and Potou sediment) were mapped to the final ASV set of the corresponding marker (*rbc*L, *rpoC1*, *psb*A, or COI‐5P) to quantify ASV presence and relative abundance. To avoid bias in abundance estimates, mapping was performed on the processed reads (barcode‐ and primer‐trimmed and length‐filtered) prior to exclusion based on average quality. Read‐to‐ASV assignment was conducted in USEARCH using the “otutab” function (Edgar 2010), which outputs the number of reads mapped to each ASV. Following Hebert et al. (2025), ASVs with ≤ 5 mapped reads in a given library were treated as absent; all others were considered present. For each library, the relative abundance of an ASV was calculated as its mapped read count divided by the total number of reads mapped to any ASV in that library.

### 2.7 ASV Taxonomic Assignment and Community Composition

Each ASV was initially assigned to a high‐level taxonomic group by performing BLASTn searches against the NCBI GenBank database through the NCBI BLAST API. Searches and extraction of the top hit for each ASV were automated with a custom Python script. ASVs identified as red algae were then subjected to more detailed taxonomic assignment at the lowest possible level. For this step, ASVs were first clustered into 98% OTUs (Operational Taxonomic Unit) and analyzed phylogenetically (as described below). BLAST results were manually inspected to confirm the reliability of the assignments and to detect possible misidentifications. For example, several COI‐5P ASVs showed misleading top BLAST hits to red algae; this issue was examined in detail using a marker‐specific phylogenetic analysis.

To visualize community composition, ASVs were grouped into broad taxonomic categories. These included Archaeplastida (red and green algae), Cryptista (cryptomonads), and the SAR supergroup, represented in our dataset by Stramenopiles (brown algae, diatoms, oomycetes, and other Stramenopiles) and Alveolata (dinoflagellates). Remaining reads were grouped as coccolithophores, choanoflagellates, amoebae, bacteria (cyanobacteria and other bacteria), animals, and unclassified sequences. For each library, category‐level read counts were obtained by summing reads from all ASVs assigned to the same taxonomic category and were then expressed as relative abundances (percentage of total mapped reads). Data aggregation, calculation, and visualization were performed in R (R Core Team 2014).

### 2.8 Red Algal Diversity Analysis

To assess whether differences in sequencing depth influenced the recovery of red algal ASV richness, we performed rarefaction and extrapolation analyses using the iNEXT package (Hsieh et al. 2016). This approach estimates expected ASV richness and evaluates the completeness of ASV recovery relative to sequencing depth within each library. We also report sample coverage (SC), a measure of within‐library coverage ranging from 0 to 1, where higher values indicate a greater probability that an additional read would belong to an ASV already detected in that library. Analyses were conducted separately for each library using abundance vectors of red algal ASVs.

Red algal ASVs were clustered into OTUs at 98% sequence similarity using CD‐HIT (Fu et al. 2012). These OTUs were used as approximate species‐level units, following the barcode‐gap threshold used in previous red algal work. This approach also reduced reliance on BLAST‐based species assignments, which were often uncertain: several ASVs showed < 98% identity to GenBank references, and some were as low as 90%, indicating potential novel or cryptic taxa not represented in current databases. Clustering at this level thus provided a consistent and conservative framework for assessing species‐level diversity.

For taxonomic identification, OTUs were included in a phylogenetic analysis alongside representative reference sequences. As COI‐5P yielded no red algal ASVs, downstream analyses were restricted to the plastid markers *rbc*L, *rpoC1*, and *psb*A. Reference sequences were obtained from the database of Zhan et al. (2020), which provides plastid gene alignments derived from representative Rhodophyta plastid genomes spanning major orders and families. In addition, for each OTU, the top BLASTn hit from earlier NCBI GenBank searches was included to capture the closest possible match. The combined reference dataset (Table S2), consisting of sequences from Zhan et al. (2020) and GenBank BLAST hits, was used to infer phylogenies, allowing OTU identification to the lowest possible taxonomic level and visualization of their placement within the broader red algal phylogeny.

Alignments were generated in MEGA 12 (Kumar et al. 2024). For each of the three alignments, the best‐fitting model of molecular evolution was determined with jModelTest 2 (Darriba et al. 2012). In all cases, the General Time Reversible model with gamma‐distributed rates and a proportion of invariant sites (GTR + G + I) was selected and applied in subsequent analyses. Maximum‐likelihood phylogenies were inferred in MEGA 12, and branch support was assessed with 1000 bootstrap replicates. Resulting phylogenies were parsed for OTU‐focused visualization by pruning reference sequences and collapsing selected internal nodes, then visualized using the Python package ETE3 (Huerta‐Cepas et al. 2016).

OTUs were assigned at the species level when they clustered with a single closest reference sequence or within a well‐supported species clade (bootstrap support ≥ 98%) and showed ≥ 98% sequence identity to that reference, following the identity threshold used by Zhan et al. (2020). OTUs were assigned at the genus level when supported by > 70% bootstrap and > 83% sequence identity. OTUs with lower similarity values or ambiguous placements were assigned at the order or class level based on their position in the phylogenetic topology.

After OTU clustering, OTU read counts were obtained by summing reads from all ASVs within each OTU and were expressed as relative abundances, calculated as a percentage of total red algal reads in each sample. Taxonomic labels from the phylogenetic assignment were then used to annotate OTUs in the diversity and abundance visualizations. Data processing and visualization were performed in R (R Core Team 2014).

We characterized the recovered red algal assemblage to determine which functional groups of red algae were represented in the workflow, including whether CCA were recovered. OTUs were classified into one of seven algal functional groups: five marine (CCA, non‐CCA encrusting—a single OTU identified to the order Hildenbrandiales, which is entirely non‐CCA encrusting—frondose, turf, and unicellular), one freshwater (OTUs from the exclusively freshwater orders Thoreales and Batrachospermales), and one unknown group (OTUs identified only to order or class level, when the taxon includes multiple functional groups and a single category could not be assigned). Functional group read counts were calculated using the same procedure as for OTUs, by summing reads from all ASVs within each group and normalizing them as a percentage of total red algal reads in each sample (relative abundance). The presence and relative abundance of each functional group were then visualized in each sample (Yongan water and sediment, Potou water and sediment). Calculations and visualizations were carried out in R (R Core Team 2014).

## 3 Results

### 3.1 Ecological Background of Two Surveyed Sites

Environmental parameters are summarized in Table S3 and visualized in Figure 1F. Differences between locations are summarized descriptively using Hedges' *g* effect sizes, with values above 0.8 conventionally considered large. The largest contrast (|*g*| > 2) was observed for oxidation–reduction potential (ORP) in August, when values were higher at Potou. Additionally, very large effects (|*g*| > 1) were observed for pH and dissolved oxygen (DO) in April, and for temperature and salinity in August, with values higher at Yongan. A large effect was also observed for pH in August (|*g*| > 0.8), with values again higher at Yongan (Table S4). In April, the higher pH and DO values observed at Yongan may be consistent with increased photosynthetic activity during the spring algal reproductive season in the algal‐reef habitat. In August, the higher temperature and salinity at Yongan may reflect evaporation in the shallow reef waters during summer. Conversely, lower salinity observed at Potou may be related to the nearby freshwater input noted during field sampling and is consistent with the detection of freshwater algal taxa in Potou water eDNA. ORP was higher at Potou in August, suggesting more oxidizing water‐column conditions at the time of measurement; however, the seasonal crossover (ORP higher at Yongan in April and higher at Potou in August; Figure 1F) indicates that additional observations are required to interpret this pattern. Overall, these patterns are consistent with more enclosed and photosynthetically active reef waters at Yongan, particularly in spring, and greater freshwater influence at Potou. However, greater temporal replication would be required to determine whether they represent consistent site‐level characteristics.

### 3.2 Sequencing Statistics and ASV Generation

The Nanopore sequencing output ranged from approximately 44,000 reads for the *rbc*L amplicon from the Yongan water sample to 1,300,000 reads for the *rpoC1* amplicon from the Potou water sample (Table S5). After read processing, average read quality improved (Figure S1A), and 23%–58% of reads were retained across samples (Figure S1B; Table S5). Overall, *rpoC1* amplicons produced the highest number of reads, while *psb*A yielded the fewest (Figure S1B; Table S5). Most reads were retained after selecting for barcode presence, with the largest proportion of discarded reads removed due to low quality (Figure S1B).

After combining filtered reads for each amplicon, a total of 401,731 (*rbc*L), 1,203,543 (*rpoC1*), 140,514 (*psb*A), and 194,502 (COI‐5P) reads were used to generate ASVs. This resulted in 94, 568, 131, and 70 ASVs with maximum sequence lengths of 778, 661, 276, and 251 bp for *rbc*L, *rpoC1*, *psb*A, and COI‐5P, respectively (lengths may differ slightly due to insertions or deletions that can shorten or extend individual sequences). The higher number of *rpoC1* ASVs may be explained by two factors: (1) a greater number of initial reads in the libraries, and (2) higher genetic diversity, resulting in multiple variants per species. The negative control library yielded 452 reads, corresponding to 0.04%–1.03% of those obtained from the environmental libraries. When screened against all barcode sequences used in the sequencing run, 429 reads (94.9%) matched one of the barcode sequences. However, after residual barcode removal and trimming 25 bp from both the 5′ and 3′ ends, all retained reads were shorter than 200 bp and were discarded by the first length filter. Therefore, no negative control reads were retained for ASV inference or taxonomic assignment (Table S5).

### 3.3 Taxonomic Composition and Marker Specificity

**FIGURE 2:**
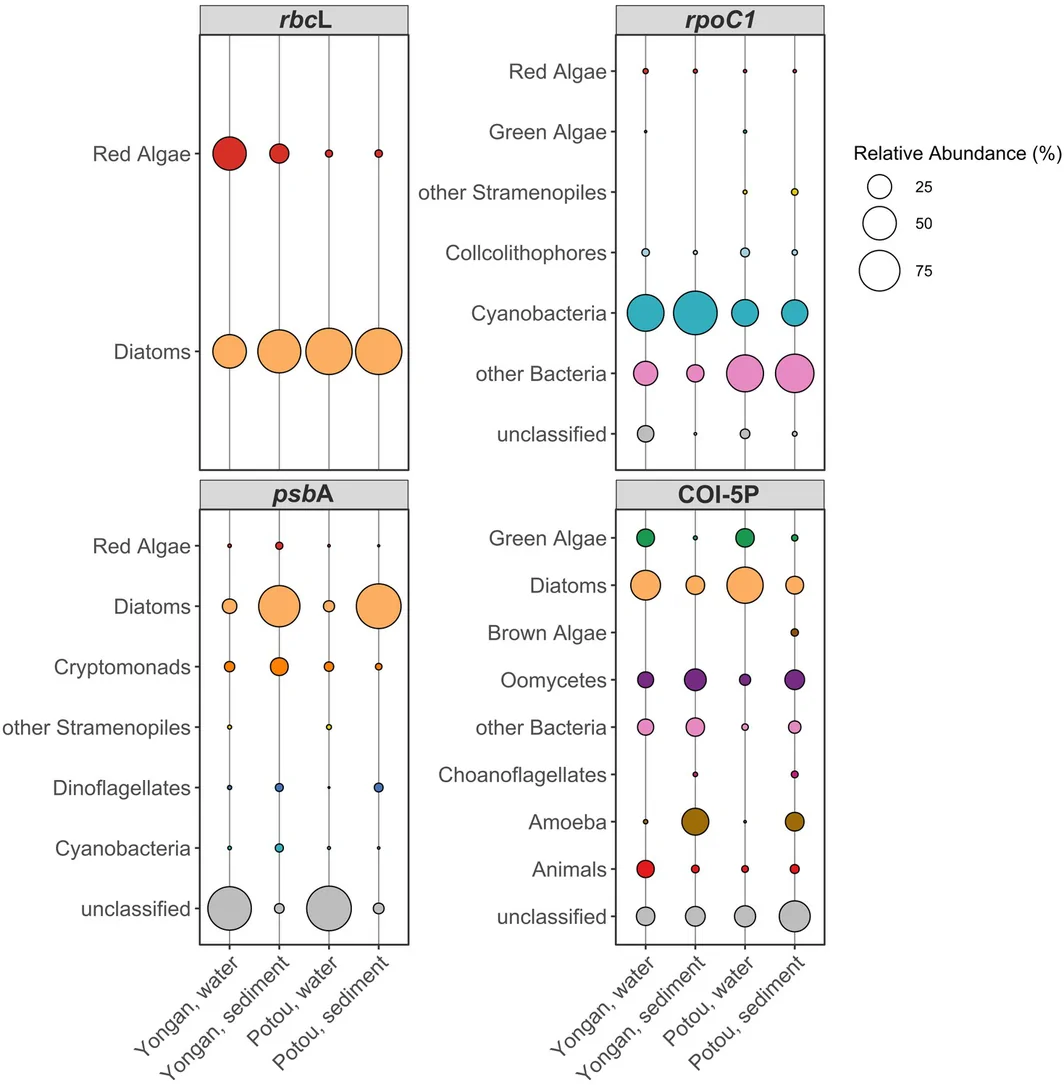
Relative abundance, expressed as a percentage of total reads, for ASVs identified to a higher taxonomic level across four datasets. Each plot represents a different genetic marker: *rbc*L, *rpoC1*, *psb*A, and COI‐5P. ASVs were classified based on the following taxonomic groups: Archaeplastida (red and green algae), Cryptista (cryptomonads), the SAR supergroup, represented by Stramenopiles (brown algae, diatoms, oomycetes, and other Stramenopiles) and Alveolata (dinoflagellates), coccolithophores, choanoflagellates, amoebae, bacteria (cyanobacteria and other bacteria), animals, and unclassified sequences. For *rbc*L, red algal relative abundance values were 49.59%, 15.44%, 1.62%, 1.81%; for *rpoC1*, values were 0.73%, 0.38%, 0.20%, 0.21%; and for *psb*A, values were 0.24%, 1.65%, 0.08%, 0.04% in Yongan water, Yongan sediment, Potou water, and Potou sediment samples, respectively.

*rbc*L recovered only red algae (Archaeplastida) and diatoms (SAR; Stramenopiles), whereas the other markers recovered a broader taxonomic range. *rpoC1* recovered red and green algae (Archaeplastida) but was dominated by nontarget ASVs assigned to unclassified Stramenopiles (SAR), coccolithophores, and especially bacteria. *psb*A recovered red algae (Archaeplastida) and cryptomonads (Cryptista), but much of the recovered signal came from SAR groups, particularly diatoms, but also dinoflagellates and unclassified Stramenopiles, with additional bacterial reads. COI‐5P detected no red algae; instead, it captured green algae (Archaeplastida) and, within SAR, diatoms, brown algae, and oomycetes; and bacteria, choanoflagellates, amoebae, and animals, reflecting its wide eukaryotic reach. The absence of red algal COI‐5P ASVs was further investigated using a marker‐specific phylogenetic analysis, as presented in the extended results of Supporting Information and Figure S2. Together, these patterns highlight strong marker‐specific biases and support combining markers to minimize false absences. *rbc*L yielded no unclassified reads, consistent with either better reference database coverage for this marker or its narrower taxonomic scope (red algae and diatoms). All plastid markers (*rbc*L, *rpoC1*, and *psb*A) showed higher relative abundances of red algal reads in the Yongan libraries than in the Potou libraries (Figure 2). Top BLAST hits, alignment statistics, and taxonomic groupings for all ASVs are provided in Table S6.

### 3.4 Marker‐Specific Recovery of Red Algal Diversity and Phylogeny

A total of 11 red algal ASVs were identified for *rbc*L (772 bp), 66 for *rpoC1* (568 bp), 21 for *psb*A (267 bp), and none for COI‐5P. The final sequence length is shorter than the original amplicon because length trimming was applied during bioinformatic processing to ensure comparability. Using a 98% identity threshold as a proxy for species‐level classification, we recovered 10 OTUs from *rbc*L, 34 from *rpoC1*, and 16 from *psb*A. Of the three plastid markers, *rpoC1* exhibited the highest OTU richness, followed by *psb*A and *rbc*L.

**FIGURE 3:**
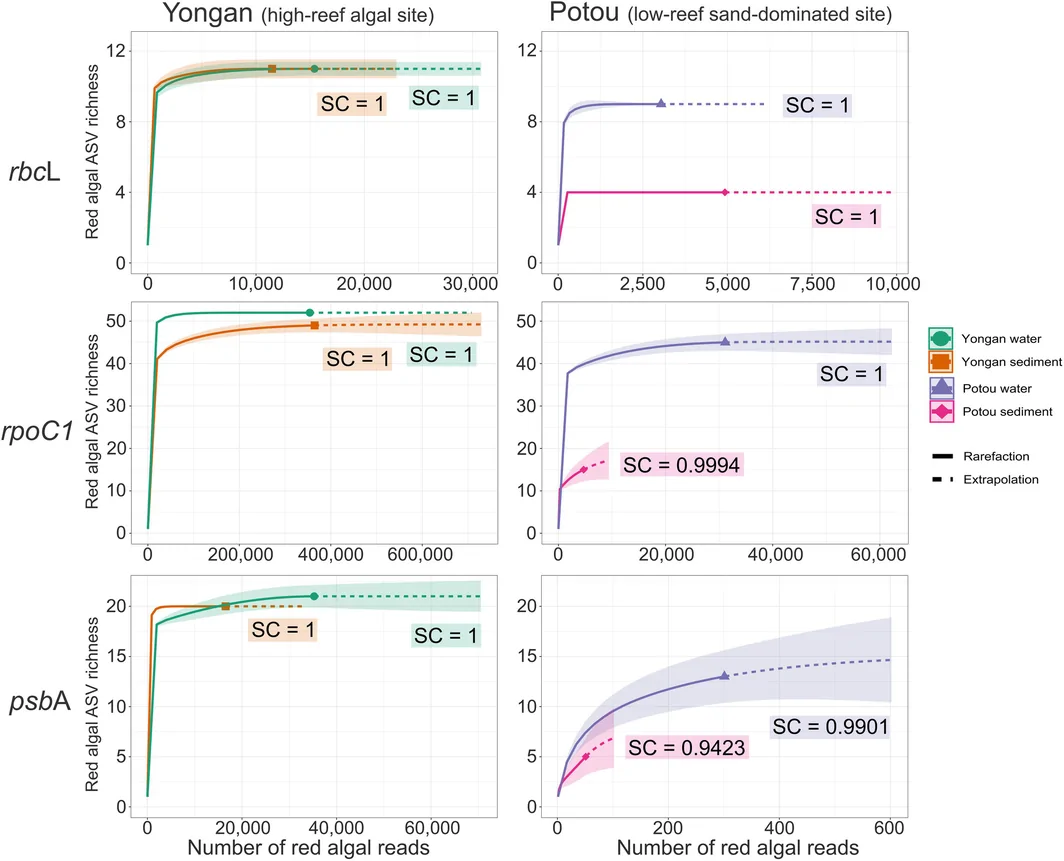
Rarefaction and extrapolation curves for red algal ASV richness generated with iNEXT. Curves are shown for *rbc*L, *rpoC*1, and *psb*A across water and sediment libraries from Yongan and Potou. Solid lines indicate rarefaction and dashed lines indicate extrapolation; points indicate observed libraries; shaded bands indicate 95% confidence intervals. SC = sample coverage, the estimated 0–1 probability that an additional read from the same library would belong to an ASV already detected.

Rarefaction analysis indicated that sequencing depth was generally sufficient to recover most of the detectable red algal ASV richness within all *rbc*L libraries and all Yongan libraries, as shown by curves that reached a plateau and estimated sample coverage values (SC = 1; Figure 3). At Potou, within‐library sample coverage varied among markers. The *rpoC1* water library reached a clear plateau (SC = 1), whereas the *rpoC1* sediment library showed slightly lower coverage (SC = 0.9994) and its curve did not fully saturate. The least complete recovery was observed for *psb*A at Potou, where both water and sediment showed incomplete saturation, lower sample coverage values (SC = 0.9901 and 0.9423, respectively), and very few mapped red algal reads (301 and 51, respectively). These results describe sequencing coverage within individual libraries and do not indicate whether the full red algal diversity of either site was captured by the field sampling design.

**FIGURE 4:**
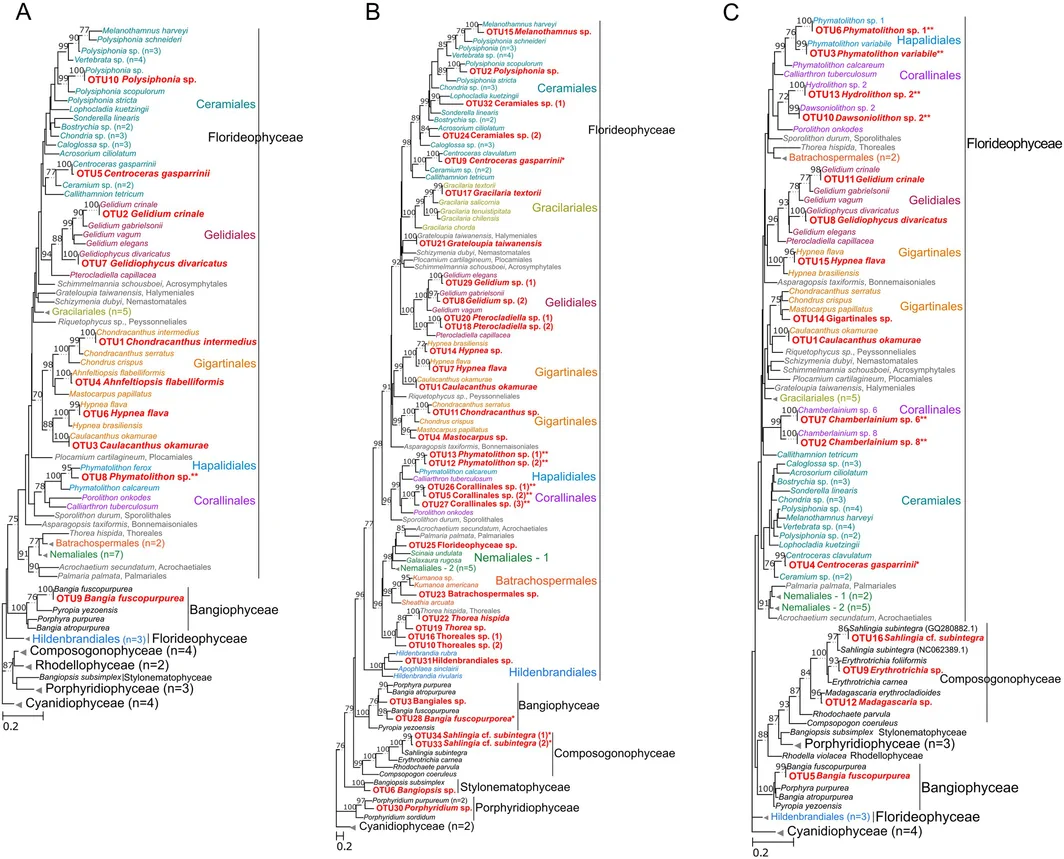
Maximum‐likelihood phylogenies of Rhodophyta from plastid genes *rbc*L (A), *rpoC1* (B), and *psb*A (C). Trees include representatives of Rhodophyta together with OTUs (Operational Taxonomic Units) recovered in this study; Cyanidiophyceae serves as the outgroup. Species names for OTUs were assigned from these phylogenies following the criteria described in the text. *: Species‐level IDs inferred from cross‐marker evidence. *rpoC1* OTU09 and *psb*A OTU04 were assigned to *C. gasparrinii* and not *C. clavulatum* based on *rbc*L, for which sequences of both species are available; for *rpoC1*/*psb*A, only the *C. clavulatum* sequence is available. For *rpoC1* OTU28/33/34 and *psb*A OTU15, single‐marker criteria would limit IDs to genus, but concordant species calls across markers justified reporting the species. **: The taxonomic identification was further confirmed by the genus presence in TAR (Zhan et al. 2022).

Phylogenetic analyses (Figure 4) were largely congruent across the three markers and with established red algal relationships, with a few exceptions. At a higher taxonomic level, *rpoC1* recovered the expected placement of major lineages, whereas *rbc*L and *psb*A showed less resolution in this respect. For example, Hildenbrandiales were grouped within Florideophyceae only in the *rpoC1* tree. Within the Florideophyceae, order‐level relationships were largely stable, but localized discordances differed among markers. Gigartinales was recovered as two clades in *rpoC1* and *psb*A, whereas *rbc*L retained a single clade, while Corallinales was nonmonophyletic only in *rpoC1*, forming a clade with the other CCA order, Hapalidiales. In *psb*A, Corallinales additionally included a distinct monophyletic *Chamberlainium* clade, likely because this lineage was represented only in the *psb*A dataset. Nemaliales was poorly resolved in *rpoC1* and *psb*A but showed greater stability in *rbc*L. Overall, the three markers showed complementary phylogenetic performance: *rbc*L provided the most stable order‐level relationships within Florideophyceae, *rpoC1* provided the best resolution at higher phylogenetic levels, and *psb*A contributed additional CCA recovery but showed greater order‐level discordance.

Regarding OTUs, the three markers recovered partly overlapping sets of taxa, with marker‐specific differences. For *rbc*L (Figure 4A), OTUs were concentrated within Florideophyceae and spanned four orders, with most in Gigartinales. CCA lineages were represented only by Hapalidiales, with none in Corallinales. One additional OTU fell in the basal red algal class Bangiophyceae. For *rpoC1* (Figure 4B), OTUs were more broadly distributed within Florideophyceae, spanning 10 orders, with one OTU unassigned to order within Florideophyceae. Five CCA OTUs were recovered across Hapalidiales and Corallinales. Additional OTUs occurred in Bangiophyceae, Compsopogonophyceae, Stylonematophyceae, and Porphyridiophyceae. For *psb*A (Figure 4C), OTUs spanned five Florideophyceae orders. These included six CCA OTUs across Hapalidiales and Corallinales, with further representation in Bangiophyceae and Compsopogonophyceae. Across all three markers, nearly all OTUs were assigned at least to a genus, and most to a species. Ten *rpoC1* OTUs and one *psb*A OTU were resolved only to order, and a single *rpoC1* OTU only to the class Florideophyceae.

**FIGURE 5:**
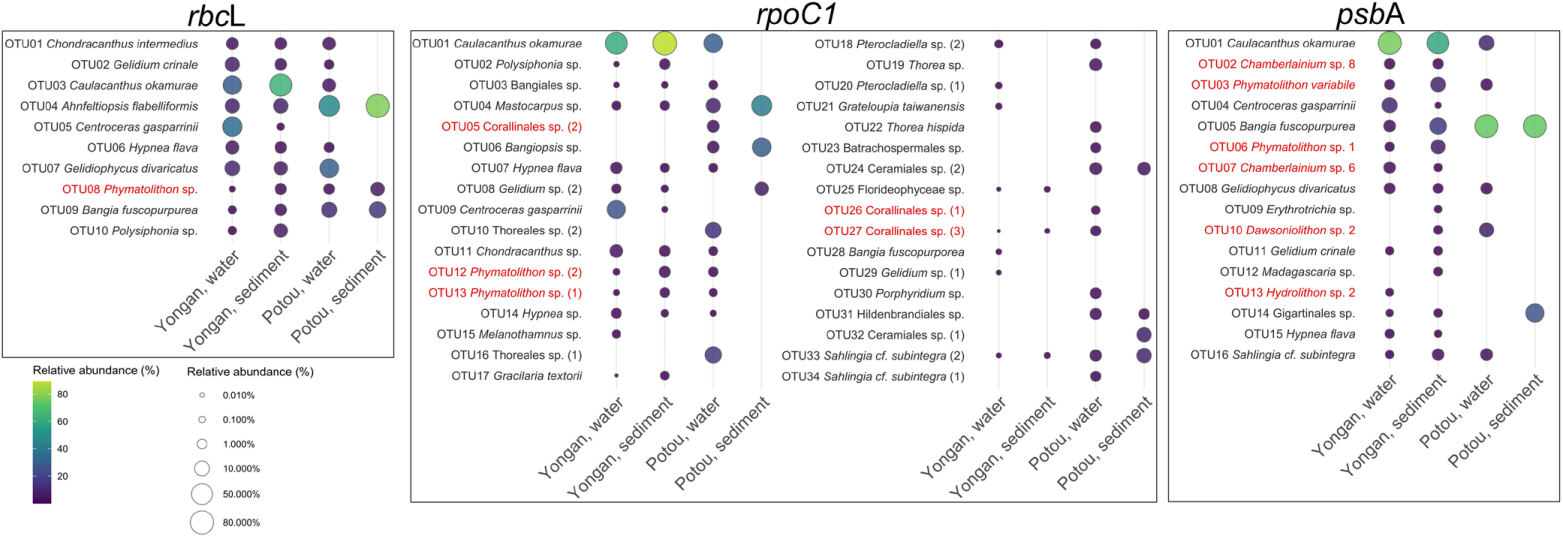
Relative abundance (expressed as percentage of total red algal reads) for 98% OTUs (Operational Taxonomic Units) corresponding to red algae across four datasets. Each plot represents a different genetic marker: *rbc*L, *rpoC1*, and *psb*A (no red algal reads were retrieved for COI‐5P). OTUs were identified based on the phylogenies shown in Figure 4. OTUs corresponding to CCA species are in red.

Across markers, the most abundant OTUs by read count were assigned to *Caulacanthus okamurae*, which was especially abundant in the Yongan libraries but was not detected in the Potou sediment library. In the Potou libraries, the most abundant assigned species differed by marker: *Ahnfeltiopsis flabelliformis* for *rbc*L, *Mastocarpus* sp. for *rpoC1*, and *Bangia fuscopurpurea* for *psb*A; all three were also detected in the Yongan libraries but were not the top taxa (Figure 5). Most CCA OTUs were recovered with *psb*A: 6 of 16 OTUs were CCA, compared with 5 of 34 in *rpoC1* and 1 in *rbc*L. *rpoC1* captured a broader set of low‐abundance taxa that were rare or absent with the other markers (i.e., *Porphyridium* (Porphyridiophyceae) and freshwater Thoreales and Batrachospermales) (Figure 5). Overall, red algal recovery varied among markers and sampled composites, supporting the use of multiple markers to reduce marker‐specific false absences and broaden the recovered red algal assemblage.

### 3.5 Recovered Red Algal Functional Groups and Field Context

**FIGURE 6:**
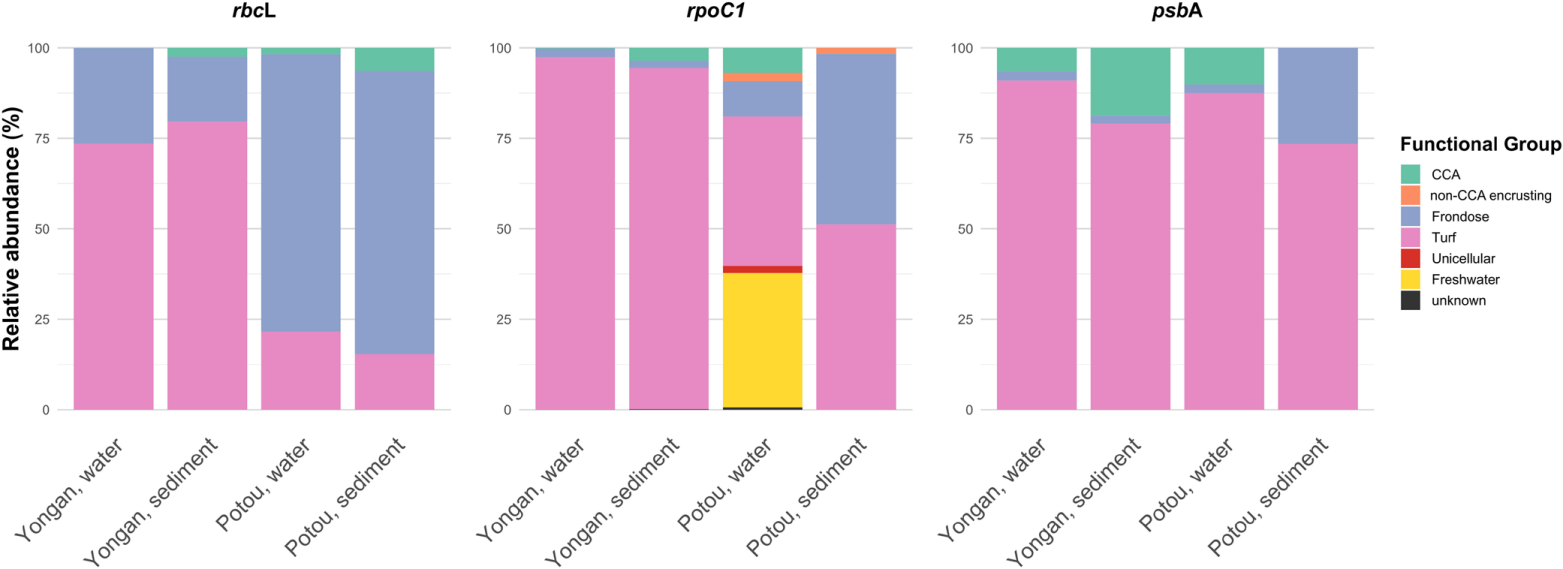
Composition of algal functional groups in the four libraries. Relative abundance (percentage of total red algal reads) for each group, defined by growth form (e.g., frondose, turf, crustose) and habitat affinity (marine vs. freshwater), is shown for the four libraries: Yongan water, Yongan sediment, Potou water, and Potou sediment.

The recovered red algal assemblage included CCA as well as several non‐CCA ones, with most retained genera classified as turf and frondose (Figure 6). Consistent with its higher OTU richness, *rpoC1* recovered the broadest range of functional groups, adding non‐CCA encrusting, unicellular taxa, and freshwater taxa. Freshwater taxa, together with a small signal of unicellular taxa, were detected only in the Potou water library, where freshwater input was also observed during field sampling (Figure S3).

Field observations provided visual context for the contrasting habitats and for several taxa recovered in the eDNA dataset. At Yongan, algal reef structures and CCA‐dominated substrate were visible (Figure S3A,B), and field photographs show *Caulacanthus okamurae*, *Chondracanthus intermedius*, and *Ahnfeltiopsis flabelliformis* from the site (Figure S3C–E). In contrast, Potou showed a sand‐dominated shore habitat with scattered rocks, no obvious CCA cover, and freshwater stream input (Figure S3F–H).

## 4 Discussion

This study demonstrates the potential of environmental DNA (eDNA) combined with Oxford Nanopore sequencing for red algal biodiversity assessment in coastal environments. Using a multilocus ONT workflow and a marker‐specific bioinformatic pipeline, we optimized extraction, library preparation, and read processing for red algal targets and showed that long‐amplicon eDNA metabarcoding can recover informative red algal assemblages from both water and sediment. A practical advantage of ONT was its ability to accommodate commonly used red algal markers with different amplicon lengths, ranging from approximately 350 to 850 bp, within a single pooled sequencing run. This flexibility allowed markers with complementary taxonomic and phylogenetic performance to be compared within the same eDNA workflow. The workflow was evaluated through a pilot application at a high‐reef site within the TAR and a nearby low‐reef sandy shore. As emphasized by Ip et al. (2026), optimization of eDNA metabarcoding workflows is inherently case‐specific, and no single best‐practice approach can avoid trade‐offs among cost, turnaround time, and detection sensitivity. eDNA metabarcoding is increasingly used to assess aquatic biodiversity as an efficient, scalable alternative to morpho‐taxonomic surveys, but its accuracy depends strongly on primer choice and the completeness of reference databases (DiBattista et al. 2020; Altermatt et al. 2025; Chen et al. 2025; Iacaruso et al. 2026). Within this framework, our study identifies *rpoC1* as the most effective single marker for capturing red algal diversity and shows that combining it with *psb*A and *rbc*L provides broader coverage of red algal lineages. Because water and sediment collected from three positions at each site were pooled before sequencing, the resulting composites did not provide independent biological replication. Comparisons between Yongan and Potou and between water and sediment should be therefore interpreted as descriptive patterns specific to the sampled composites, rather than as general differences between habitat types. Broader ecological inference would require independent spatial and temporal replication.

Our study indicates that the primers were not fully specific to red algae. All four primer sets amplified a broad range of nontarget taxa. COI‐5P, despite being widely used for specimen‐based red algal barcoding, failed to detect any red algae in our pilot workflow. In contrast, a recent work using the 313 bp Leray fragment of COI, together with 18S‐V4, recovered a broad macroalgal assemblage (Leray et al. 2013; Brooks et al. 2026). This contrast suggests that the lack of red algal detection with COI‐5P in our dataset may be assay‐specific rather than reflecting an inherent limitation of COI for macroalgal eDNA metabarcoding. More broadly, nontarget amplification in complex coastal settings may limit the recovery of low‐abundance target DNA. Although the plastid markers recovered red algae, red algal reads were consistently a minority of total reads. Nevertheless, sequencing depth provided sufficient red algal reads for downstream analyses in most libraries, despite substantial nontarget amplification. The low‐depth *psb*A libraries from Potou, however, should be interpreted with caution. Non‐target composition differed among plastid markers, reflecting evolutionary history and primer–template interactions. For *rbc*L and *psb*A, diatoms dominated the nontarget pool, consistent with the secondary endosymbiotic red algal origin of diatom plastids (Moustafa et al. 2009). This result is likely reinforced by the ecological dominance of diatoms in coastal phytoplankton or benthic communities (De Vargas et al. 2015; Malviya et al. 2016). In contrast, *rpoC1* libraries were dominated by bacterial reads, including cyanobacteria, plausibly reflecting cross‐amplification linked to the cyanobacterial ancestry of plastids (Keeling 2010). Given the high abundance of bacteria in marine and sedimentary environments (Whitman et al. 1998), even limited cross‐amplification can markedly reduce red algal recovery. Together, these patterns show that nontarget amplification reflects predictable biological constraints that should be explicitly considered when selecting markers for red algal eDNA surveys.

The rarefaction analysis indicates that sequencing depth was generally sufficient to recover the detectable red algal ASV richness within most libraries. Complete estimated sample coverage was obtained for all *rbc*L libraries and all Yongan libraries. Among the remaining libraries, *rpoC1* Potou sediment and *psb*A Potou water showed slightly incomplete saturation but still high estimated coverage, suggesting that additional sequencing would recover relatively few further ASVs. The clearest exception was *psb*A Potou sediment, where the low number of mapped red algal reads and lower sample coverage suggest that additional sequencing could potentially recover further low‐abundance ASVs. Thus, although the lowest‐depth Potou libraries should be interpreted with caution, incomplete sequencing depth does not appear to be the primary explanation for the observed marker‐specific recovery patterns. These results concern sequencing completeness within the analyzed libraries and do not indicate whether the full red algal diversity of either site was captured, which would require independent spatial and temporal replication.

Despite substantial nontarget amplification, red algal diversity was recovered across major phylogenetic lineages. The greater OTU richness recovered by *rpoC1* likely reflects a combination of higher intrinsic sequence variability, greater effective read depth, and marker‐specific primer performance. Phylogenetically, *rpoC1* also provided the best resolution at higher phylogenetic levels, broadly consistent with Zhan et al. (2020), who found that its gene tree more closely matched the phylogeny inferred from complete plastid genomes, although *rbc*L showed greater stability for some order‐level relationships within Florideophyceae. Across the combined phylogenies from the three plastid markers, OTUs were broadly distributed rather than concentrated within a few clades, indicating that the multilocus ONT eDNA workflow recovered a broad phylogenetic range of red algae within the sampled composites. Although *rpoC1* retrieved more red algal OTUs overall, a much smaller proportion could be identified to species level compared with the other markers, reflecting the poorer taxonomic coverage of *rpoC1* in reference databases. The central role of reference databases in eDNA‐based biodiversity assessments is well established, and gaps in taxonomic coverage can limit identification rates (Chen et al. 2025; Iacaruso et al. 2026). Consistent with these previous studies, our study shows that marker performance depends not only on primer design but also on the completeness and resolution of the reference database. Furthermore, some of the closest matches in the *rpoC1*, *rbc*L, and *psb*A databases came from studies in our focal area and/or were confirmed by them, highlighting the importance of building local, marker‐specific databases.

eDNA is often reported to achieve higher detection rates, species richness, and occurrence records than traditional methods (Iacaruso et al. 2026). In our study, however, CCA recovery was lower than in our prior resource‐intensive specimen‐based surveys at the same site, which used in situ collection of CCA and *psb*A DNA barcoding to delimit cryptic OTUs (Zhan et al. 2022). Within our eDNA data, *psb*A was the most effective marker for CCA, yet it still recovered fewer CCA OTUs at Yongan (6) than the 17 OTUs reported previously from specimen‐based surveys, including 14 detected in spring (Zhan et al. 2022). This contrast is unlikely to be driven mainly by seasonality, as both studies sampled during spring and CCA assemblages at the TAR have been shown to be temporally stable across seasons (Zhan et al. 2022). More broadly, eDNA recovery can be influenced by both biological and methodological factors. Organismal traits and biological shedding dynamics, including the release of cells, tissues, and extracellular DNA into the environment, can affect eDNA recovery, as shown for taxa such as ticks, which can be visually abundant yet shed little detectable DNA (Iacaruso et al. 2024). In our case, the lower CCA recovery may reflect a combination of organismal biology and methodological differences, including sampling intensity and design, sample type, extraction and amplification efficiency, primer bias, reference database coverage, and bioinformatic filtering. In particular, the present study used a single eDNA sampling event, whereas the previous specimen‐based study included three spring surveys. Together, these observations indicate that, in this pilot dataset, targeted multilocus ONT eDNA recovered only part of the known CCA assemblage at Yongan, and that future applications should include more replicates and calibration against specimen‐based surveys.

Despite targeting CCA, our eDNA data were dominated by turf and frondose macroalgal reads across markers. This pattern differed from previous benthic surveys at Yongan, where CCA dominated reef cover and frondose algae were relatively minor components of the substrate (Zhan et al. 2022). The high abundance of turf taxa such as *Caulacanthus okamurae* and *Centroceras gasparrinii* in our eDNA data may reflect not only their presence in the habitat but also differences in DNA release associated with organismal traits, potentially making these soft‐bodied taxa more detectable than heavily calcified CCA crusts. Turf algae are small and filamentous, forming low mats on the substrate, whereas frondose algae are larger, with leaf‐like blades and branched thalli. Both groups can be productive and heavily grazed by invertebrates (Hatcher 1990). Turf mats, in particular, can exhibit high biomass production and rapid turnover (Miller et al. 2009). Although the eDNA workflow described here did not recover CCA as extensively as expected, it may be well suited to detecting turf and frondose macroalgae. This is consistent with Borer et al. (2025), who showed that multilocus DNA metabarcoding can effectively characterize turf‐ and frondose‐dominated macroalgal communities.

In this study, we observed that marker selection also influenced the recovered assemblage. The CCA‐targeted *psb*A marker was comparatively more effective at recovering CCA signal, consistent with its design for Corallinophycidae (Anglès d'Auriac et al. 2019), whereas *rpoC1* recovered a broader set of red algal lineages, including freshwater taxa (e.g., Batrachospermales and Thoreales) at Potou, consistent with nearby stream input. The *rbc*L marker recovered CCA, turf, and foliose red algae, but fewer CCA than *psb*A. Together, these marker‐specific patterns indicate that *psb*A is useful for targeted CCA recovery, whereas *rpoC1* and *rbc*L broaden the recovered red algal assemblage. Thus, no single marker can fully represent the red algal diversity detected in this study, supporting the use of multilocus strategies for future red algal eDNA surveys.

Finally, sedimentary eDNA may serve as a useful tool for exploring macroalgal contributions to sedimentary organic carbon. This includes testing whether higher reef cover is associated with enhanced retention of red algal material in sediments. Experimental work in other blue carbon habitats has shown that sedimentary eDNA can covary with macrophyte‐derived carbon and stable isotope signatures (Ortega et al. 2020; Chang et al. 2024; Hung et al. 2024), suggesting that similar approaches could be applied to red algal systems. Although the present study was not designed to assess carbon retention, the optimized sediment eDNA extraction and analytical workflow developed here provides a methodological foundation for future studies addressing this question. Such applications would require more spatial, temporal, and technical replication, together with marker‐specific experiments to establish the quantitative relationship between red algal eDNA abundance and red algal organic carbon in sediments.

## Author Contributions

**Silvia Fontana:** data curation (equal), formal analysis (equal), investigation (equal), methodology (equal), software (equal), validation (equal), visualization (equal), writing – original draft (equal), writing – review and editing (equal). **Wun‐Ruei Chang:** data curation (equal), investigation (equal), methodology (equal). **Shao‐Lun Liu:** conceptualization (equal), funding acquisition (equal), investigation (equal), supervision (equal), writing – original draft (equal), writing – review and editing (equal).

## Conflicts of Interest

The authors declare no conflicts of interest.

## Supporting information


**Figure S1:** Nanopore sequencing yield and read processing results across 16 libraries, each representing a unique combination of location (Yongan or Potou), environment (water or sediment), and genetic marker (*rbc*L, *rpoC1*, *psb*A, or COI‐5P). (A) Bars show the average read quality of the original reads, and reads after processing, before applying a quality filter. (B) Bars show the number of total reads, barcode‐assigned reads, and reads that passed the quality filter.
**Figure S2:** Maximum‐likelihood phylogeny based on COI‐5P sequences, including representative Rhodophyta reference sequences, all COI‐5P ASVs recovered in this study, and their closest NCBI GenBank BLASTn hits. *Asterisk indicates putative nontarget amplification.
**Figure S3:** Photographs of the two sampling sites and selected red algae observed in the field. (A–E) Yongan, the high‐reef site. (A) View of the Yongan shore, with visible algal reef structures. (B) Close‐up of reef substrate dominated by crustose coralline algae (CCA). (C–E) Red algae observed in the field and also recovered in the eDNA analysis: (C) *Caulacanthus okamurae*, (D) *Chondracanthus intermedius*, and (E) *Ahnfeltiopsis flabelliformis*. (F–H) Potou, the low‐reef, sand‐dominated site. (F) View of the Potou shore, characterized by sand and scattered rocks rather than developed reef structures. (G) Close‐up of a rock at Potou, showing no obvious CCA cover. (H) View of the Potou shore showing freshwater stream input, which provides possible context for the detection of freshwater red algal taxa in the Potou water library.

**Table S1:** Information about primers used for eDNA.
**Table S2:** Reference dataset used for phylogenetic analysis of plastid loci.
**Table S3:** Environmental parameters measured in situ at the two sampling locations (Yongan and Potou), in April 2023 (during biological sampling) and again in August 2023. For each site and month, values are mean ± SD based on three sampling dates (*n* = 3). Water temperature, salinity (PSU), pH, oxidation–reduction potential (ORP), and Dissolved Oxygen (DO) are shown.
**Table S4:** Hedges' *g* effect sizes (95% confidence intervals, CI) comparing Yongan vs. Potou, calculated in two separate analyses: one using April measurements and one using August measurements. Positive *g* indicates higher values at Yongan (relative to Potou); negative *g* indicates higher values at Potou.
**Table S5:** Nanopore sequencing yield and read‐processing results for the 16 environmental libraries and the negative control.
**Table S6:** Top BLASTn hit summary for all ASVs across markers (rbcL, rpoC1, psbA, COI‐5P).

## Data Availability

All relevant source code required to reproduce the analyses in this study is available at https://github.com/silviafontana/edna‐metabarcoding‐nanopore‐redalgae. Raw read data are available in the NCBI Sequence Read Archive (SRA) under BioProject PRJNA1418491. Final ASV and OTU sequence datasets are available on Figshare at https://doi.org/10.6084/m9.figshare.31268128.
