# Supplementary material for: A Multilocus Nanopore eDNA Workflow for Red Algal Diversity Assessment in an Algal Reef System
Source: Ecol Evol. 2026 Aug 23;16(8):e74209. doi: 10.1002/ece3.74209 (PMC13500228; doi:10.1002/ece3.74209)
Supplement: Supplementary file 1 [file ECE3-16-e74209-s002.docx]

**SUPPORTING INFORMATION**

**Extended Results**

**COI-5P Performance and Absence of Red Algal ASVs**

BLASTn searches of the 70 COI-5P ASVs recovered from environmental libraries indicated that five ASVs had red algae as their top GenBank hit, four of which were closely related and mapped to the same reference. However, preliminary inspection suggested that these sequences did not cluster with other red algal COI-5P sequences.

To investigate this pattern, a COI-5P phylogeny was constructed, including representative Rhodophyta COI-5P sequences from Saunders (2005), Robba et al. (2006), Yang et al. (2015) and Yang et al. (2016), spanning major red algal orders and families. In addition, for each ASV, the top BLASTn hit from GenBank was included. Alignments were generated in MEGA 12, the best-fitting model (GTR+G) was selected using jModelTest 2, and maximum-likelihood phylogenies were inferred in MEGA 12 with adaptive bootstrap. Phylogenies were visualized using ETE3.

The COI-5P phylogeny **(Figure S2**) showed that none of the environmental COI-5P ASVs clustered within the well-supported Rhodophyta clade, which included the basal genus *Cyanidium*. Instead, ASVs whose top BLAST hits were assigned to red algae were positioned outside this clade and grouped with amoebozoan sequences, indicating incorrect taxonomic assignment in the reference database. These results indicate that the COI-5P primers amplified non-target templates, most likely epiphytic organisms and/or intracellular bacteria present in the samples, rather than red algal mitochondrial DNA. This mis-amplification likely also explains why several ASVs initially identified as animals clustered with bacteria or brown algae in the phylogeny. Together, these results confirm that COI-5P failed to recover genuine red algal sequences in this study and possibly amplified non-target organisms present in or on the samples, such as epiphytes or associated bacteria.

**SUPPORTING INFORMATION REFERENCES**

Robba, L., Russell, S. J., Barker, G. L., and Brodie, J. (2006). Assessing the use of the mitochondrial cox1 marker for use in DNA barcoding of red algae (Rhodophyta). *Am. J. Bot.* 93, 1101–1108. doi: 10.3732/ajb.93.8.1101

Saunders, G. W. (2005). Applying DNA barcoding to red macroalgae: a preliminary appraisal holds promise for future applications. *Philos. Trans. R. Soc. B: Biol. Sci.* 360, 1879–1888. doi: 10.1098/rstb.2005.1719

Yang, E. C., Boo, S. M., Bhattacharya, D., Saunders, G. W., Knoll, A. H., Fredericq, S., et al. (2016). Divergence time estimates and the evolution of major lineages in the florideophyte red algae. *Sci. Rep.* 6, 21361. doi: 10.1038/srep21361

Yang, E. C., Kim, K. M., Kim, S. Y., Lee, J., Boo, G. H., Lee, J.-H., et al. (2015). Highly conserved mitochondrial genomes among multicellular red algae of the Florideophyceae. *Genome Biol. Evol.* 7, 2394–2406. doi: 10.1093/gbe/evv147

**SUPPLEMENTAL FIGURES**

**Figure S1.** Nanopore sequencing yield and read processing results across 16 libraries, each representing a unique combination of location (Yongan or Potou), environment (water or sediment), and genetic marker (*rbc*L, *rpoC1*, *psb*A, or COI-5P). (A) Bars show the average read quality of the original reads, and reads after processing, before applying a quality filter. (B) Bars show the number of total reads, barcode-assigned reads, and reads that passed the quality filter.

**Figure S2.** Maximum-likelihood phylogeny based on COI-5P sequences, including representative Rhodophyta reference sequences, all COI-5P ASVs recovered in this study, and their closest NCBI GenBank BLASTn hits. * indicates putative non-target amplification.

**Figure S3.** Photographs of the two sampling sites and selected red algae observed in the field. (A–E) Yongan, the high-reef site. (A) View of the Yongan shore, with visible algal reef structures. (B) Close-up of reef substrate dominated by crustose coralline algae (CCA). (C–E) Red algae observed in the field and also recovered in the eDNA analysis: (C) *Caulacanthus okamurae*, (D) *Chondracanthus intermedius*, and (E) *Ahnfeltiopsis flabelliformis*. (F–H) Potou, the low-reef, sand-dominated site. (F) View of the Potou shore, characterized by sand and scattered rocks rather than developed reef structures. (G) Close-up of a rock at Potou, showing no obvious CCA cover. (H) View of the Potou shore showing freshwater stream input, which provides possible context for the detection of freshwater red algal taxa in the Potou water library.
